# Selection against individuals from genetic introgression of escaped farmed salmon in a natural population of Atlantic salmon

**DOI:** 10.1111/eva.13213

**Published:** 2021-03-27

**Authors:** Sebastian Wacker, Tonje Aronsen, Sten Karlsson, Ola Ugedal, Ola H. Diserud, Eva M. Ulvan, Kjetil Hindar, Tor F. Næsje

**Affiliations:** ^1^ Norwegian Institute for Nature Research Trondheim Norway

**Keywords:** aquaculture, Atlantic salmon, farmed salmon, genetic introgression, *Salmo salar*, survival

## Abstract

The viability of wild Atlantic salmon populations is threatened by genetic introgression from escaped farmed salmon. Farmed Atlantic salmon are genetically improved for important commercial traits and a life in captivity but are poorly adapted to the natural environment. The rate of gene flow from escaped farmed to wild salmon depends on their spawning success and on offspring survival at various life stages. We here investigate relative survival of introgressed juvenile Atlantic salmon (parr) in a river in northern Norway. The studied population has experienced genetic introgression from farmed salmon for about four generations (20 years). We followed two cohorts of parr from the year of hatching (0+) to the age of 2 years (2+). Farmed genetic introgression was quantified at the individual level and on a continuous scale using diagnostic SNPs. Population‐level genetic introgression decreased from 0+ to 2+ by 64% (2011 cohort) and 37% (2013 cohort). This change was driven by a 70% (2011 cohort) and 49% (2013 cohort) lower survival from age 0+ to 2+ in introgressed parr compared to parr of wild origin. Our observations show that there is natural selection against genetic introgression with a potential cost of lower productivity.

## 1 INTRODUCTION

Domesticated animals that escape from captivity or are released intentionally may hybridize with wild conspecifics, leading to unidirectional gene flow into wild populations. Examples of genetic introgression from domesticated animals into wild populations include mammals (Anderson et al., 2019; Kidd et al., 2009), birds (Brisbin & Peterson, 2007; Wu et al., 2020), fish (Letourneau et al., 2018) and insects (Seabra et al., 2019). Genetic introgression from domesticated animals alters the gene pool of wild populations and may constrain their viability and evolutionary potential (Glover et al., 2017; Naylor et al., 2005). Farmed domesticated animals are adapted to a captive environment and selected for characteristics that are of commercial importance. The same characteristics may reduce survival and reproductive success in the natural environment (Araki et al., 2007; Bertolotti et al., 2020). Domesticated animals may also originate from a limited set of founder populations and from a geographical range that does not reflect the genetic diversity of the species (Hindar et al., 1991). Reduced genetic diversity and non‐native origin are also commonly found in captive‐bred animals that are intentionally released into the environment for the purpose of stocking wild populations (Kitada, 2018; Letourneau et al., 2018). Due to domestication selection and the origin of founder populations, genetic introgression from escaped farmed animals and intentionally released domesticated animals is expected to reduce genetic diversity and to interfere with local adaption of wild populations.

The fast‐growing aquaculture industry commonly involves farming of fish species outside their natural distribution and farming of highly domesticated fish species (Bostock et al., 2010; Naylor et al., 2001). Escaped farmed fish threaten native species through the introduction of invasive species and through hybridization between domesticated individuals and wild conspecifics (Araki & Schmid, 2010). Among the best‐documented examples of genetic introgression from farmed domesticated fish into wild populations is Atlantic salmon (*Salmo salar*) (Forseth et al., 2017; Glover et al., 2017; Karlsson et al., 2016; Wringe et al., 2018). Farmed Atlantic salmon in Norway originate from several wild founder populations from western Norway and have been selected for traits that are favourable in aquaculture since the 1970s (Gjedrem & Baranski, 2009; Gjedrem et al., 1991). They hold lower genetic variation compared to wild Atlantic salmon and differ in fundamental life‐history traits such as growth and maturation (Bolstad et al., 2017; Glover et al., 2017). Farmed Atlantic salmon kept in aquaculture outnumber their wild conspecifics 1000‐fold and escape events occur frequently (Fiske et al., 2006; Glover et al., 2019, 2020).

Escaped farmed Atlantic salmon may enter rivers and hybridize with wild Atlantic salmon, leading to unidirectional gene flow and genetic introgression (Glover et al., 2013; Karlsson et al., 2016). Hybrid and farmed offspring are able to survive to maturity in the wild and to return to freshwater for spawning (Fleming et al., 2000; McGinnity et al., 2003). Genetic introgression from escapees is thereby carried over to future generations and manifested in wild populations (Glover et al., 2013; Karlsson et al., 2016). Such farmed genetic introgression has been found in many geographic regions where wild Atlantic salmon co‐occur with Atlantic salmon farming, including Canada, Ireland and Norway (Glover et al., 2017). There was large spatial and temporal variation in the incidence of escaped farmed salmon in rivers across Norway over a 25‐year period (1989–2013), with average incidences ranging from ca. 8%–29% across geographical regions and with high incidences during the early 1990s and the early 2000s (Diserud et al., 2019). Hybridization of escaped farmed salmon with wild Atlantic salmon has resulted in an average level of farmed genetic introgression of 6.4% (range 0%–42%) in 109 rivers across Norway (Karlsson et al., 2016). These estimates are from adult salmon sampled after having spent their entire life in the wild. At the juvenile stages, the level of introgression is expected to be higher, but few comparisons exist (Karlsson et al., 2016).

Hybrids of farmed and wild Atlantic salmon are poorly adapted to the natural environment (Bolstad et al., 2017) and show lower survival and reproductive success than wild conspecifics (Fleming et al., 2000; McGinnity et al., 2003). Hybridization may thereby substantially reduce population‐level fitness of wild Atlantic salmon populations. At the same time, reduced survival and reproductive success of hybrids may limit genetic introgression into wild populations (Glover et al., 2017; Hindar et al., 2006), as found for stocking of brook charr (*Salvelinus fontinalis*) (Letourneau et al., 2018) and for hybridization between native westslope cutthroat trout (*Oncorhynchus clarkii lewisi*) and invasive rainbow trout (*O*.* mykiss*) (Kovach et al., 2016). Knowledge of survival and reproductive success of farmed hybrids is therefore important for the prediction of both population‐level fitness and genetic introgression in wild Atlantic salmon.

The early survival of hybrid Atlantic salmon from eggs to smolt in the wild has previously been studied in field experiments. Farmed and wild Atlantic salmon were allowed to interact and spawn freely in experimental rivers (Fleming et al., 2000), or eggs from crossings were planted into experimental rivers (McGinnity et al., 2003; Skaala et al., 2019). Juveniles sampled at later stages were genetically assigned to farmed and wild parents. Studies that quantified total survival in freshwater from eggs to out‐migrating smolt uniformly reported a reduced survival of farmed and hybrid individuals (Fleming et al., 2000; McGinnity et al., 2003; Skaala et al., 2012, 2019). Field experiments that quantified survival from age 0+ to out‐migrating smolt found variable survival of farmed and hybrid parr in Ireland (McGinnity et al., 1997, 2003), but little variation between groups in Norway (Fleming et al., 2000). A recent study used diagnostic SNPs to estimate the abundance of farmed and hybrid Atlantic salmon parr in a range of rivers in Canada, after a single large aquaculture escape (Sylvester et al., 2019; Wringe et al., 2018). From age 0+ to 2+, there was a reduction in the relative abundance of farmed parr and hybrids (Sylvester et al., 2019; Wringe et al., 2018). In summary, field studies uniformly reported decreased survival of hybrid and farmed offspring during the entire freshwater stage (eggs to smolt), while the evidence was mixed for parr survival (Fleming et al., 2000; McGinnity et al., 2003; Sylvester et al., 2019).

Earlier field studies on the survival of farmed and hybrid individuals in freshwater have focussed on first‐generation offspring of farmed Atlantic salmon and their crossing with wild fish. Genetic introgression from escaped farmed salmon over many generations is expected to result in offspring of various hybrid classes. Field studies in Ireland found reduced parr survival (0+ to out‐migrating smolt) for farmed and first‐generation hybrids, while second‐generation hybrids and second‐generation backcrosses between hybrids and farmed or wild fishes had reduced survival at the egg stage but not as parr (McGinnity et al., 2003). Earlier studies also focussed on high proportions of farmed and hybrid offspring (ca. 25%–75%) and on scenarios of single large‐scale introgression events (Fleming et al., 2000; McGinnity et al., 2003; Skaala et al., 2019; Wringe et al., 2018). The relative survival of hybrid parr may depend on whether they primarily compete with hybrid parr or with wild parr. Farmed parr show higher levels of aggression than wild parr (Einum & Fleming, 1997), and under constant density, the presence of farmed parr, but not the presence of wild parr, has been found to reduce the survival of wild parr (Robertsen et al., 2019; Sundt‐Hansen et al., 2015). Knowledge of the survival of hybrid parr under moderate levels of genetic introgression is also important because the negative effects of genetic introgression on wild populations may be more severe under low‐level introgression over prolonged time than under rare large‐scale introgression events (Baskett et al., 2013). The relative survival of farmed and hybrid Atlantic salmon parr has not been studied in rivers that have experienced genetic introgression over prolonged time and under moderate levels of genetic introgression.

Here, we study changes in genetic introgression over time in two cohorts (years of hatching) of Atlantic salmon parr in the River Alta in northern Norway. The Atlantic salmon in River Alta is part of the Barents–White Sea phylogenetic group, while all founder populations of the farmed strains are part of the Eastern Atlantic phylogenetic group (Bourret et al., 2013; Karlsson et al., 2016). Large genetic divergence between native River Alta Atlantic salmon and farmed escapees may increase maladaptation and thereby mortality of introgressed individuals (Baskett et al., 2013; Bolstad et al., 2017; Huisman & Tufto, 2012). Escaped farmed salmon have been recorded in River Alta since the late 1980s (Ugedal et al., 2016). The relative abundances of escaped farmed salmon in catches of adult spawners in the autumn ranged from 0% to 22% between 1991 and 2018 (Ugedal et al., 2016) (Table S1). The overall level of genetic introgression in parr of the studied cohorts was moderate. To study relative survival of introgressed and wild salmon in a natural population, we quantified genetic introgression in parr at the ages of 0+, 1+ and 2+ within two cohorts. We hypothesized that the level of genetic introgression would decrease as the cohort grew older. This study aims at understanding the ability of natural selection to reduce the level of genetic introgression of escaped farmed salmon. The results add new knowledge about the consequences of escaped farmed salmon in wild populations.

## 2 METHODS

### 2.1 River Alta

River Alta is an Atlantic salmon river in northern Norway (70°N 23°E) with an average discharge of 98.9 m^3^/s and an average catch of salmon of 16 tonnes per year (Ugedal et al., 2016). The River Alta has been utilized for hydroelectric generation purposes since 1987 and the outlet of the water tunnel from the power plant is located at the upper end of the salmon producing section, which is limited to the lower 50 km of the 160 km long main stem (Ugedal et al., 2008).

**FIGURE 1:**
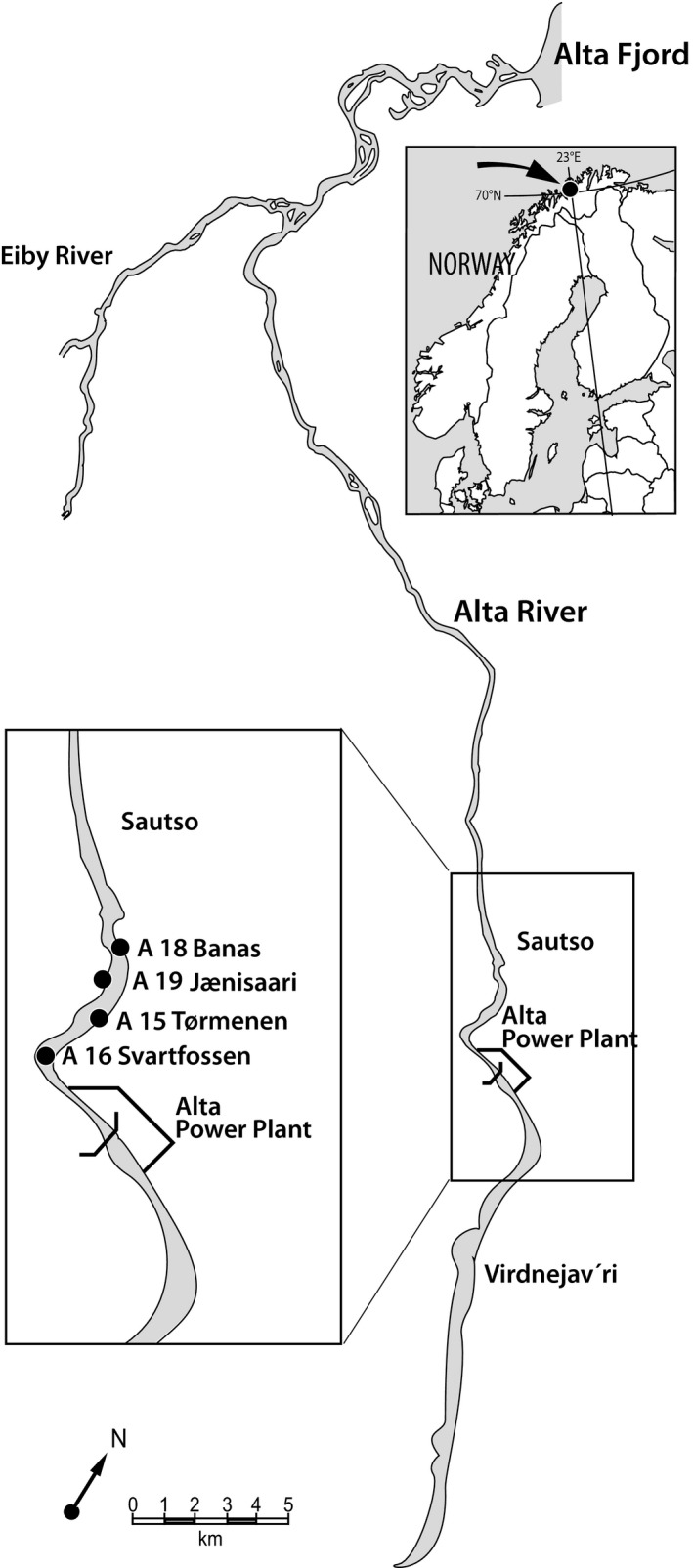
River Alta with the anadromous part of the river from the Alta Fjord to the hydropower station (Alta Power Plant). The study was conducted in the uppermost part of the river (Sautso), and samples were collected at four sampling locations (A16, A15, A19 and A18)

The population level of farmed genetic introgression, measured in adult fish and with the same methods as used in this study (described under Statistical analysis), in River Alta varied between 0% and 5.4% from 2012 to 2016 (Karlsson et al., 2016). This study was conducted in the uppermost section of River Alta, called Sautso (Figure 1), which in many years had a higher proportion of escaped farmed Atlantic salmon than the lower parts of the river (Table S1). Tagging studies have shown that escaped farmed salmon have a higher propensity than wild salmon to migrate to the upper parts of River Alta (Heggberget et al., 1996).

### 2.2 Sampling of fish

In order to study changes in the level of farmed genetic introgression within cohorts of juvenile Atlantic salmon, fish were sampled at the year of hatching (0+), 1 year after hatching (1+) and 2 years after hatching (2+). Samples were accordingly collected in 2012, 2013 and 2014 for breeding year 2011 and in 2014, 2015 and 2016 for breeding year 2013. Juveniles were collected by electrofishing at four sampling locations (Figure 1) and stored in ethanol (breeding year 2011: 0+, 1+, 2+; breeding year 2013: 0+) or frozen (breeding year 2013: 1+ and 2+). Juveniles were thereafter measured for fork length to the nearest mm, and measurements were back‐calculated to live fork length using previously established relationships (Thorstad et al., 2007). Age of juveniles was determined from readings of scales and otoliths. Sampling took place between August and October, with, in most instances, two sampling days per age class and cohort (Table 1). The habitat at sampling location A19 is not suitable for 2+ parr and only a single and no fish of that age were caught at sampling location A19 for the breeding years 2011 and 2013, respectively (Table 1). We present results for the cohorts from the breeding years 2011 and 2013. For a third cohort (breeding year 2014), juveniles were collected at the ages of 0+ and 1+ and analysed for farmed genetic introgression. The level of genetic introgression in the 2014 cohort was marginal and not statistically significant at the age of 0+ (0.5%) and 1+ (1.5%). The data were therefore not suited to test for a change in the level of genetic introgression with increasing age.

**TABLE 1:** Numbers of juvenile Atlantic salmon from two cohorts (breeding years 2011 and 2013) collected at four sampling locations in River Alta (A15, A16, A18, A19)

	2011			2013		
	0+	1+	2+	0+	1+	2+
A15	23 (0 + 23)	26 (25 + 1)	26 (11 + 15)	24 (24 + 0)	24	46 (18 + 28)
A16	26 (19 + 7)	25 (17 + 8)	24 (12 + 12)	25 (0 + 25)	29	24 (9 + 15)
A18	24 (0 + 24)	26 (26 + 0)	24 (2 + 22)	22 (22 + 0)	24	30 (14 + 16)
A19	22 (8 + 14)	23 (12 + 11)	1 (1 + 0)	22 (22 + 0)	18	0
Total	95 (27 + 68)	100 (80 + 20)	75 (26 + 49)	93 (68 + 25)	95	100 (41 + 59)

Note

Juveniles were sampled at the year of hatching (0+) and at the age of one and two years (1+ and 2+). When sampling of a given cohort and age class took place at two different occasions, numbers of fish sampled at the first and second sampling date, respectively, are given in brackets.

### 2.3 Genetic analysis

DNA was extracted from juvenile fish stored in ethanol using the Dneasy tissue kit (Qiagen) and genotyped at 81 nuclear and 15 mitochondrial SNPs using a EP1™ 96.96 Dynamic array IFCs platform (Fluidigm). Forty‐eight of the nuclear SNPs have been identified by Karlsson et al. (2011) as showing large genetic differences between Norwegian farmed and wild salmon regardless of farmed strain and wild population, and these were used for estimating wild and farmed ancestry of individual fish (Karlsson et al., 2014, 2016).

### 2.4 Estimating genetic introgression

We estimated genetic introgression with the method described by Karlsson et al. (2014). The method uses the programme STRUCTURE (Pritchard et al., 2000) to estimate the likelihood of an individual to belong to a wild salmon reference sample versus a farmed salmon reference sample. We hereafter refer to this likelihood as *P*(*Wild*) (Karlsson et al., 2014). The wild reference sample is given by historical samples collected before the onset of commercial Atlantic salmon farming. River Alta belongs to the Barents–White Sea phylogenetic group (Bourret et al., 2013) and historical samples from a range of populations belonging to this phylogenetic group were used as wild reference (Karlsson et al., 2014, 2016). Samples from the Norwegian breeding kernels for farmed salmon were used as farmed salmon reference (Karlsson et al., 2014, 2016).

Genetic introgression on the population level (proportion of the genome being of farmed origin) was estimated from individual *P*(*Wild*) estimates. Individual *P*(*wild*) estimates range from zero to one, so wild reference samples will always have an average *P*(*wild*) estimate less than one, while the farmed salmon reference sample has an average *P*(*wild*) estimate larger than zero (Karlsson et al., 2014). When estimating genetic introgression on population level, the scale must therefore be calibrated by the respective average observed *P*(*Wild*) in the wild and farmed reference samples (Karlsson et al., 2014). Historical samples from River Alta collected in 1981 and 1982 (Karlsson et al., 2016) were used as wild reference sample for calibrating the scale of population‐level genetic introgression. This procedure ensured unbiased estimation of genetic introgression for River Alta.

The power of quantifying introgression on individual and population level with the above methods has been explored in simulations (Karlsson et al., 2014). On population level, introgression was estimated with high precision; that is, the estimate was close to the simulated proportion of the genome being of farmed origin. On the individual level, introgression is estimated with larger uncertainty and *P*(*wild*) estimates for first‐generation hybrids may cover the whole range from 0 to 1 (Karlsson et al., 2014).

### 2.5 Statistical analysis

We tested whether population‐level genetic introgression was significantly larger than 0 within each cohort and age class. This was done by testing if the observed mean *P*(*Wild*) was smaller than the mean *P*(*Wild*) of the historical sample from the River Alta (Karlsson et al., 2016) with a two‐sample t test. *P*(*Wild*) estimates (proportion data varying from zero to one) were logit‐transformed before testing to achieve that transformed proportions are approximately normally distributed (Karlsson et al., 2014). Tests assumed equal variance of samples (Karlsson et al., 2014, 2016).

A linear model was used to test for a temporal change of genetic introgression within cohorts, that is an effect of age on *P*(*Wild*). The model was fitted with *P*(*Wild*) as response variable and with age (continuous variable) and cohort (factor) as explanatory variables. In graphical exploration, there was no indication for a difference in slopes among the two cohorts and the model was fitted without an interaction between age and cohort. Sampling of juveniles of a given cohort and in a given year was carried out at two dates (Table 1), with 25–70 days in between. Changes in genetic introgression within cohorts may occur over time between years and between sampling dates within years, and age was therefore entered into the model as a continuous variable. Age was included in the model as the number of days counted from August 14 (the earliest date 0+ juveniles were sampled) in the year 0+ samples of the respective cohort were collected. On this scale, the age of 0+ samples was 0–77 days, the age of 1+ samples was 365–395 days, and the age of 2+ samples was 730–800 days. *P*(*Wild*) is a likelihood estimate measured on a scale from zero to one and was logit‐transformed prior to analysis and residuals inspected for deviation from normal distribution.

We also tested for a temporal change in the proportion of introgressed individuals within cohorts, classifying individuals as introgressed or wild depending on their *P*(*wild*). In contrast to the above‐described analysis of changes in *P*(*wild*), results from this analysis can be more directly related to earlier studies on the relative survival of introgressed parr, which reported abundances and relative survival of wild and hybrid parr (Fleming et al., 2000; McGinnity et al., 2003; Wringe et al., 2018). We used a generalized linear model with a binomial distribution to test for an effect of age on the likelihood of juveniles to be of wild origin. The response variable in the model was the classification of each juvenile as wild (entered as value one) or introgressed (entered as value zero) and explanatory variables were age (continuous variable) and cohort (factor). In graphical exploration, there was no indication for a difference in slopes among the two cohorts and the model was fitted without an interaction between age and cohort. There was indication for overdispersion of the residuals and the model was therefore fitted with a quasibinomial error distribution.

We classified juveniles as introgressed that had a *P*(*Wild*) below a given threshold. The threshold was based on the *P*(*Wild*) distribution of historical samples of Atlantic salmon from the Barents–White Sea phylogenetic group in Finnmark county, Norway (*N* = 1000). Those historical samples were collected before the onset of commercial Atlantic salmon farming and did therefore not include introgressed individuals. Using a threshold from the lower end of the *P*(*wild*) distribution of those historical samples ensured that juveniles from River Alta that were classified as introgressed were unlikely to be of pure wild origin. We analysed the data using three alternative lower percentiles of the historical distribution: the 5 percentile (*P*(*Wild*) = 0.8315), the 3 percentile (*P*(*Wild*) = 0.7420) and the 1 percentile (*P*(*Wild*) = 0.5528). Because of moderate levels of genetic introgression in the studied cohorts, a large proportion of juveniles were classified as wild. The number of truly wild juveniles expected to be wrongly classified as introgressed (1%–5% depending on the threshold) was therefore relatively high compared to the number of truly introgressed juveniles and highest when using the 5 percentile threshold. When the 1 percentile was used, few juveniles were classified as introgressed, increasing uncertainty in the statistical estimation of the proportion of introgressed individuals. We therefore present results based on the 3 percentile in the main text. Results for all three considered percentiles are presented in Table S2.

Classification of juveniles into wild and introgressed was also used to calculate survival of introgressed juveniles relative to survival of wild juveniles. Relative survival of introgressed juveniles was calculated for the two cohorts separately and for the entire time period (0+ to 2+ age), as well as for the time periods from 0+ to 1+ and from 1+ to 2+ separately. Relative survival of hybrid juveniles was calculated by dividing the ratio of introgressed to wild individuals in the first sample (e.g. 0+ age) by the ratio of introgressed to wild individuals in the second sample (e.g. 2+ age).

The fact that a substantial proportion of juveniles classified as introgressed was likely truly wild renders our analysis of a temporal change in introgression within cohorts conservative, because potential differences in survival between wild and introgressed juveniles are partly masked. This effect is expected to be stronger under a higher *P*(*Wild*) threshold.

Parr mortality may be related to body size which again can be related to genetic introgression (Fleming et al., 2000; Reed et al., 2015; Solberg et al., 2013). The effect of genetic introgression on growth is a potential route for reduced survival in hybrid parr, and we therefore tested for an effect of *P*(*Wild*) on fork length. The effect was tested with separate linear models for each age class (0+, 1+, 2+). Models were fitted with fork length (continuous variable) and cohort (factor) as explanatory variables. There was no indication for a difference in slopes among the two cohorts and the model was fitted without an interaction between fork length and cohort.

## 3 RESULTS

**FIGURE 2:**
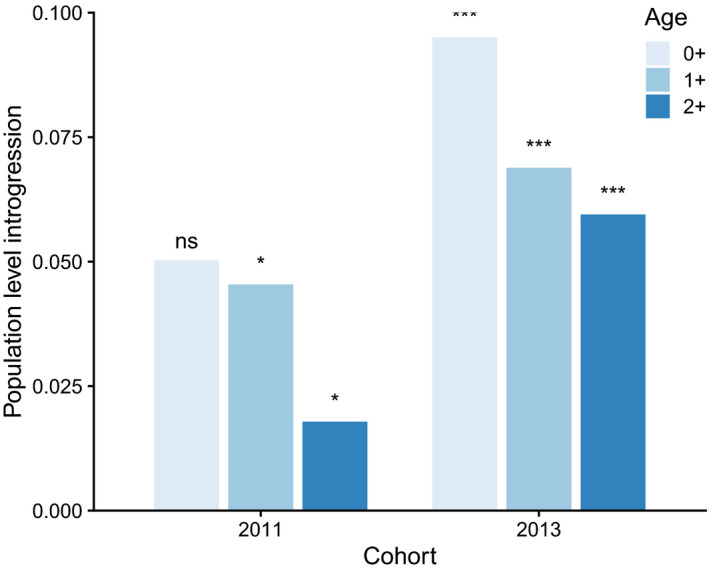
Estimated population‐level farmed introgression (proportion of the genome being of farmed origin) in juvenile Atlantic salmon from River Alta of two cohorts (2011 and 2013) at the age of 0+ to 2+. Symbols above bars indicate whether introgression was statistically significantly higher than 0 (*** *p* < 0.001, ** *p* < 0.01, * *p* < 0.05, ns *p* > 0.05)

**FIGURE 3:**
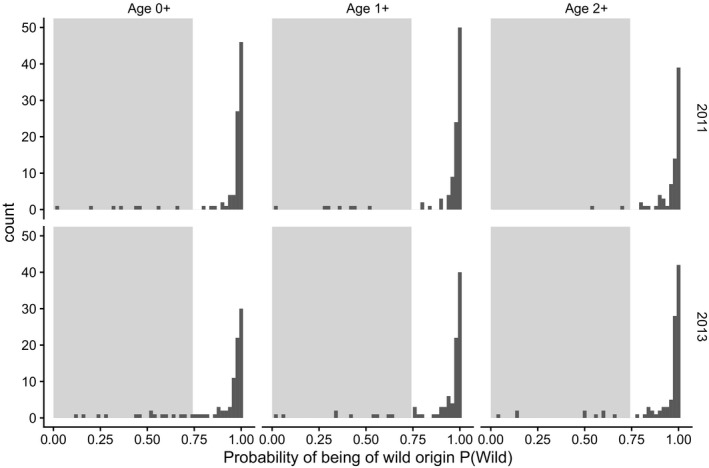
Distribution of individual *P*(*Wild*) estimates (probability of belonging to a wild reference sample) in juvenile Atlantic salmon from River Alta of two cohorts (2011 and 2013) sampled at the age of 0+, 1+ and 2+. Shaded areas indicate the range within which individuals were classified as farmed or hybrid offspring based on a *P*(*wild*) threshold of 0.7420

We detected moderate farmed genetic introgression in juvenile Atlantic salmon from River Alta. Estimated population‐level genetic introgression ranged from 0.02 to 0.10 within a cohort and age class (Figure 2) and between 3% and 16% of the sampled parr were classified as hybrids (*P*(*Wild*) < 0.7420; Figure 3). Population‐level genetic introgression was significantly higher than 0 for all age classes in the 2013 cohort (all *p* < 0.001) and for the 0+ and 1+ age classes in the 2011 cohort (both *p* < 0.05) (Figure 2).

**FIGURE 4:**
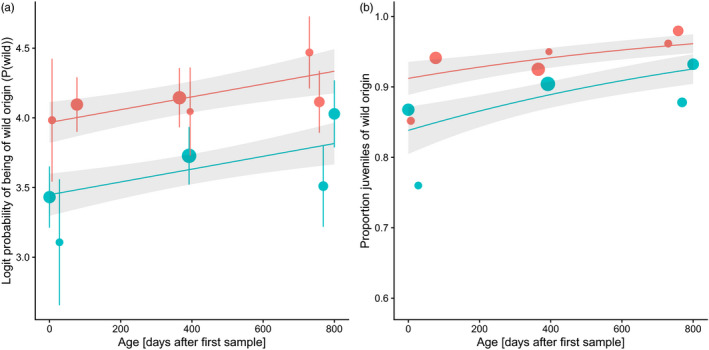
Effect of age on genetic introgression in two cohorts (red =2011, green =2013) of juvenile Atlantic salmon from River Alta. Age is given as the number of days after first sampling in the year of hatching for each cohort: 0+ (0–77 days), 1+ (365–395 days) and 2+ (730–800 days). Genetic introgression is given as (a) logit‐transformed probability of being of wild origin *P*(*Wild*) and (b) the classification of individuals as being of wild origin or as introgressed, based on their *P*(*Wild*). Points indicate means per sampling date and the size of points indicates sample size (*N* between 20 and 94 individuals). For (a) *P*(*Wild*), bars indicate one standard error. Lines indicate effects estimated in (a) a linear model and (b) a generalized linear model, with shaded areas indicating one standard error

Estimated population‐level genetic introgression decreased with the age of parr within cohorts. In the 2011 cohort, genetic introgression (the estimated proportion of the genome being of farmed origin) changed from 0.050 at 0+ age to 0.018 at 2+ age (64% reduction). In the 2013 cohort, genetic introgression changed from 0.095 at 0+ age to 0.059 at 2+ age (37% decrease) (Figure 2). We tested for a temporal change in individual *P*(*Wild*) (the probability of belonging to the wild reference sample) within cohorts and there was a statistically nonsignificant trend for an increase with parr age (slope ±SE: 0.000459 ± 0.000260 logit *P*(*wild*)*day^−1^; *F* = 3.1, *p* = 0.078; Figure 4a). Overall the level of genetic introgression was higher in the 2013 than in the 2011 cohort, with a lower *P*(*Wild*) (intercept: *F* = 11.1, *p* < 0.001; Figure 4a).

The proportion of juveniles that were classified as wild (*P*(*Wild*) ≥ 0.7420) increased with the age of parr (slope: χ^2^ = 4.8, *p* = 0.029; Figure 4b; Table S2). An overall lower proportion of juveniles was classified as wild in the 2011 cohort than in the 2013 cohort (intercept: χ^2^ = 5.2, *p* = 0.023; Figure 4b). The estimated temporal increase in the proportion of individuals classified as wild was stronger when a lower *P*(*wild*) threshold was used and weaker when a higher *P*(*wild*) threshold was used (Table S2).

The survival of introgressed juveniles from 0+ to 2+, relative to the survival rate of wild parr, was estimated at 0.30 (0+ to 1+: 0.82; 1+ to 2+: 0.36) and 0.51 (0+ to 1+: 0.55; 1+ to 2+: 0.93) in the 2011 and 2013 cohorts, respectively. The proportion of juveniles that were classified as introgressed varied widely among sampling localities, but decreased with age in most sampling localities within breeding years (Figure S1).

**FIGURE 5:**
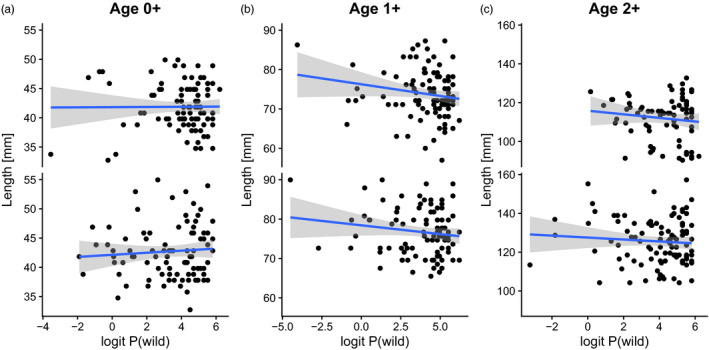
Relationship between genetic introgression (logit *P*(*Wild*)) and fork length [mm] in two cohorts (2011 upper panel and 2013 lower panel) of Atlantic salmon parr sampled in River Alta at the age of 0+ (a), 1+ (b) and 2+ (c). Blue lines indicate relationships estimated by linear regression, and grey shades indicate 95% confidence intervals. Note that scales on both axes differ among panels

Parr length was significantly negatively associated with *P*(*Wild*); that is, introgressed parr were larger than wild parr, at the age of 1+ (*F* = 5.0, *p* = 0.027; Figure 5b), and there was a statistically nonsignificant trend for such a relationship at the age of 2+ (*F* = 3.6, *p* = 0.06; Figure 5c), but not at the age of 0+ (*F* = 6.7, *p* = 0.55; Figure 5a). At the age of 1+, there was an estimated decrease of 7.2% in fork length between the fish of lowest *P*(*Wild*) (2011 cohort: 78.3 mm) and the individual of highest *P*(*Wild*) (2011 cohort: 72.7 mm). In the model of length at 1+ age, cohort and logit *P*(*Wild*) together explained approximately 8% of the variation in fork length.

## 4 DISCUSSION

Farmed genetic introgression decreased over the first 2 years after hatching in two cohorts of Atlantic salmon. The results show that introgressed parr had a lower survival than wild parr in River Alta. Survival of introgressed parr in the wild has previously been studied in Canada after a major escape event (Wringe et al., 2018) and in field experiments in Norway and Ireland (Fleming et al., 2000; McGinnity et al., 2003; Skaala et al., 2012, 2019). In line with our results, introgressed parr had a lower survival than wild parr in Canada (Sylvester et al., 2019; Wringe et al., 2018). Temporal changes in the relative abundance of introgressed parr differed widely in strength and direction among the thirteen rivers studied, but on average the relative abundance of farmed and hybrid parr was halved from the age of 0+ to 2+ (Sylvester et al., 2019). Reduced survival of farmed parr and first‐generation hybrids was also found in Ireland (0+ to out‐migrating smolt), after eggs from crossings of farmed and wild Atlantic salmon were planted into an experimental river (McGinnity et al., 1997, 2003; Reed et al., 2015). Fleming et al. (2000) released wild and farmed adult Atlantic salmon into an experimental river in Norway and quantified breeding success and offspring survival. Breeding success and early survival were lower for farmed than for wild fish, but there was no evidence for reduced survival of introgressed offspring in the parr stage (0+ in autumn to out‐migrating smolt). Our results add to previous evidence of reduced survival of farmed and hybrid parr from Canada and Ireland.

We found reduced relative survival of introgressed parr in a river with moderate levels of genetic introgression. In the studied cohorts, population‐level genetic introgression was only 5%–10% at the age of 0+, with 9%–16% of parr detected as introgressed. Despite moderate levels of genetic introgression, there was a substantial decrease in introgression from 0+ to 2+ (37%–64%) and the relative survival of introgressed parr was 0.30–0.51 across the same time period. There was considerable uncertainty in estimating those changes, as expected when analysing low rates of introgression and low proportions of introgressed parr. With a sample size of approximately 300 individuals per cohort, we detected significant introgression in both cohorts and a significant reduction in introgression with age, but the statistical significance was around the 0.05 acceptance threshold in several tests. Despite those uncertainties, our study shows that introgressed Atlantic salmon parr show reduced survival under moderate levels of introgression. Reduced survival of introgressed parr under moderate levels of introgression is a finding that complements earlier studies that quantified the survival of hybrid parr under higher relative abundances in field experiments (Fleming et al., 2000: >25%; McGinnity et al., 2003: 75%) and in Canadian rivers (median relative abundance 50%; Wringe et al., 2018). Our results also show that the survival of hybrid parr is reduced in rivers experiencing moderate levels of genetic introgression over prolonged time periods. This result is important for predicting the effect of genetic introgression on the viability of wild populations, which is expected to be stronger under constant low‐level genetic introgression than under rare events of strong genetic introgression (Baskett et al., 2013). Together with previous studies, our results show reduced survival of introgressed parr across a range of levels of genetic introgression.

Our study considered introgression resulting after about 20 years of varying abundances of escaped farmed salmon (Table S1) (Diserud et al., 2019). Introgressed parr were therefore expected to belong to various hybrid classes, resulting from backcrosses between farmed, wild, and hybrid parents over several generations. Previous experimental studies followed survival of first‐generation (Fleming et al., 2000; McGinnity et al., 1997) or first‐ and second‐generation hybrids (McGinnity et al., 2003). This was also primarily the case in the observational study in Canada, where a large‐scale escape event affected previously little introgressed rivers (Sylvester et al., 2019; Wringe et al., 2018). This may affect results because the effects of genetic introgression on survival and fitness vary among the different backcross types, and variation does not necessarily follow an additive manner (Debes et al., 2013; McGinnity et al., 2003; Wringe et al., 2018). McGinnity et al. (2003) found reduced survival for farmed parr and first‐generation hybrids, but not for second‐generation hybrids or backcrosses, which experienced increased relative mortality only at the egg stage. Our study did not detail survival rates for specific hybrid and backcross types. Instead, our results show that the total level of genetic introgression within cohorts decreased in parr over 2 years in a population that had experienced interbreeding with escaped farmed salmon over several generations.

Variation in the relative survival of introgressed parr may ultimately have a strong effect on the rate of gene flow into wild populations. In models based on experimental studies from Norway and Ireland, 0+ to smolt was the life stage at which differences in survival rates among experiments affected genetic introgression the most (Hindar et al., 2006). The relative survival of introgressed parr in our study was at the lower end of the range considered in those models, implying a reduction in population‐level introgression under a given abundance of farmed escapees (Hindar et al., 2006). Relative survival of introgressed parr was also found to largely affect the rate of gene flow in models based on observations made after a large‐scale escape of farmed salmon in Canada (Sylvester et al., 2019). Variation in the effects of introgression on survival has also been observed at other life stages, including early development and from smolt to returning adults (McGinnity et al., 2003; Robertsen et al., 2019; Sundt‐Hansen et al., 2015). In consequence, the relative contribution of those effects to the relative fitness of introgressed Atlantic salmon varied. Together, those effects are likely to contribute to the elusive factors explaining the relationship between abundances of escaped farmed Atlantic salmon and resulting genetic introgression across Norwegian rivers (Karlsson et al., 2016).

The negative impact of genetic introgression may not only depend on the rate of gene flow but also on genetic divergence between farmed and wild strains (Baskett et al., 2013; Castellani et al., 2015; Glover et al., 2017; Huisman & Tufto, 2012). Larger genetic divergence is expected to result in larger genetic impact on locally adapted wild populations. At the same time, larger genetic divergence may increase mortality of introgressed Atlantic salmon and thereby slow down the rate of gene flow. The negative impact may therefore be more severe under moderate genetic divergence (Baskett et al., 2013; Huisman & Tufto, 2012). Genetic divergence is largest when farmed strains originate from other phylogenetic groups than the local wild populations belong to. This is the case for Irish populations, but also for wild populations in northern Norway, which are part of the Barents–White Sea phylogenetic group, while all founder populations of the farmed strains are part of the Eastern Atlantic phylogenetic group (Bourret et al., 2013; Karlsson et al., 2016). Genetic introgression has therefore a potentially large genetic impact in River Alta and other populations of the Barents‐White Sea phylogenetic group. Bolstad et al. (2017) found a significant effect of genetic introgression on sea‐age and size at maturity and that this effect was different, and for some comparisons larger, in the Barents–White Sea phylogenetic group compared to the effects in the Eastern Atlantic phylogenetic group. The high relative mortality of introgressed parr found in this study is in line with the expectation that high genetic divergence leads to more pronounced maladaptation and mortality of introgressed individuals.

Possible mechanisms for the effect of introgression on survival are related to the faster growth rate of introgressed Atlantic salmon parr. Differences in growth rate between farmed and wild parr are substantial under farming conditions (Glover et al., 2009, 2018), but reduced under semi‐natural (Einum & Fleming, 1997; Sundt‐Hansen et al., 2015) and natural conditions (Fleming et al., 2000; Reed et al., 2015; Solberg et al., 2013). We found a moderate increase in body length (ca. 8%) with the level of introgression at age 1+ and 2+, but introgression explained only a small part of size variation. Increased growth rates under natural conditions may be an overproportional investment into growth at the cost of fat reserves, which may in turn increase winter mortality (Finstad et al., 2004). Overproportional investment into growth may be particularly costly in terms of survival in northern regions, where local populations show adaptations to long winters (Finstad et al., 2010; Finstad & Forseth, 2006) and due to the negative energy balance fat reserves may be important for their survival throughout the winter (Næsje et al., 2006). Farmed Atlantic salmon parr may be maladapted to such northern conditions as a result of selection for commercially important traits and because of their origin from western Norwegian populations (Gjedrem & Baranski, 2009).

An alternative mechanism for the effect of introgression on survival is predation, as farmed parr have been shown to be more risk‐prone than wild parr (Einum & Fleming, 1997). Recently, an experimental study found that predation of brown trout (*S*.* trutta*) on Atlantic salmon juveniles could explain the markedly lower survival of farmed and hybrid offspring, whereas the same experimental groups had similar survival in the absence of trout (Solberg et al., 2020).

Temporal changes in genetic introgression may not only have been affected by the relative survival of introgressed parr but also by movement of parr between the studied upper part of River Alta and lower parts of the river. Larger introgressed parr may be superior in ecological competition with wild parr and have been found to displace wild parr under semi‐natural conditions (Sundt‐Hansen et al., 2015) and in natural rivers (McGinnity et al., 2003). Displacement of wild parr by introgressed parr cannot be excluded in our study, but would render our analysis conservative, given that survival of displaced wild parr would result in a stronger decrease in population‐level introgression within cohorts.

## 5 CONFLICT OF INTEREST

None declared.

## Supporting information

Supplementary Material

## Data Availability

The data that support the findings of this study are openly available in Dryad at https://doi.org/10.5061/dryad.9kd51c5gm.
