# Supplementary material for: Selection against individuals from genetic introgression of escaped farmed salmon in a natural population of Atlantic salmon
Source: Evol Appl. 2021 Mar 27;14(5):1450–60. doi: 10.1111/eva.13213 (PMC8127704; doi:10.1111/eva.13213)
Supplement: Supplementary file 1 — Supplementary Material [file EVA-14-1450-s001.pdf]

Supplementary material for:

Selection against individuals from genetic introgression of escaped farmed salmon in a natural population of Atlantic salmon

Sebastian Wacker, Tonje Aronsen, Sten Karlsson, Ola Ugedal, Ola H. Diserud, Eva M. Ulvan, Kjetil Hindar, Tor F. Næsje

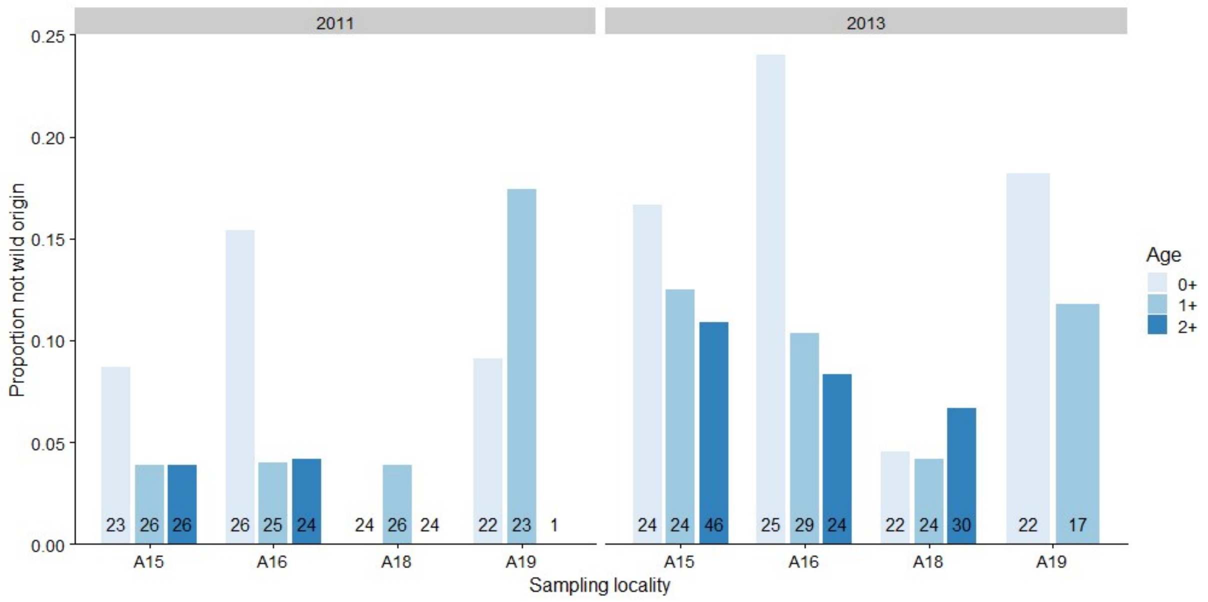

Figure S1. Proportion of fish that were classified as introgressed (P(Wild)<0.7420) in dependence of sampling locality (A15, A16, A18, A19). Sample size inside bars.

Table S1. Incidence of escaped farmed salmon caught in River Alta in the autumn of the years 2009-2019. Numbers of individuals (N), numbers of escaped farmed salmon (N farmed) and relative abundance (% farmed) are reported for the uppermost part ("Sautso") and the lower parts of the river. Data originally reported in (Ugedal et al. 2016, Ugedal et al. 2017, 2018, 2019, Skoglund et al. 2020).

Year	Locality	N	N farmed	% farmed
2009	Sautso	109	5	5
2010	Sautso	158	22	14
2011	Sautso	76	9	12
	lower parts	91	15	17
2013	Sautso	45	16	36
	lower parts	93	12	13
2014	Sautso	69	13	19
	lower parts	138	12	9
2015	Sautso	19	0	0
	lower parts	155	3	2
2016	Sautso	32	0	0
	lower parts	155	2	1
2017	Sautso	32	0	0
	lower parts	139	1	<1
2018	Sautso	--	--	--
	lower parts	63	2	1
2019	Sautso	13	0	0
	lower parts	50	1	<1

Table S2. Relative survival of genetically introgressed juvenile Atlantic salmon of two cohorts (2011 and 2013) from River Alta. Individuals are categorised as introgressed and wild based on three alternative *P(Wild)* thresholds (5-percentile from historical samples: *P(Wild)*=0.8315; 3-percentile: *P(Wild)*=0.7420; 1-percentile: *P(Wild)*=0.5528). The survival of introgressed juveniles is given relative to a survival of 1 for wild juveniles and as annual survival rates (0+ to 1+; 1+ to 2+) and bi-annual survival rate (0+ to 2+). Estimates (± S.E.) from generalised linear models on the effect of cohort (intercept) and age (slope; change in proportion wild per 100 days age) are given on logit scale.

	Relative survival introgressed			Model on change in proportion wild			
	0+ to 1+	1+ to 2+	0+ to 2+	intercept	*P*	slope	*P*
P(Wild)<0.8315							
2011	0.95	0.72	0.68	2.09 ± 0.26	0.010	0.084 ± 0.044	0.055
2013	0.68	0.71	0.48	1.39 ± 0.23			
P(Wild)<0.7420							
2011	0.82	0.36	0.30	2.34 ± 0.29	0.023	0.109 ± 0.051	0.029
2013	0.55	0.93	0.51	1.65 ± 0.25			
P(Wild)<0.5528							
2011	0.95	0.18	0.17	2.43 ± 0.32	0.440	0.122 ± 0.061	0.040
2013	0.64	0.77	0.49	2.16 ± 0.30			

Skoglund, S., T. F. Næsje, G. Østborg, and L. Saksgård. 2020. Innslag av rømt oppdrettslaks i Altaelva og Repparfjordelva i 2019. 1734, Norsk institutt for naturforskning (NINA).

Ugedal, O., T. F. Næsje, L. M. Saksgård, and E. B. Thorstad. 2016. Fiskebiologiske undersøkelser i Altaelva. Samlerapport for 2011- 2015. 1265.
Ugedal, O., L. M. Saksgård, T. F. Næsje, and E. B. Thorstad. 2017. Fiskebiologiske undersøkelser i Altaelva i 2016. 74, Norsk institutt for naturforskning (NINA).
Ugedal, O., L. M. Saksgård, T. F. Næsje, and E. B. Thorstad. 2018. Fiskebiologiske undersøkelser i Altaelva i 2017. 1515, Norsk institutt for naturfoskning (NINA).
Ugedal, O., L. M. Saksgård, T. F. Næsje, and E. B. Thorstad. 2019. Fiskebiologiske undersøkelser i Altaelva i 2018. 1681, Norsk institutt for naturforskning (NINA).
